# Visualization of deglutition and gastroesophageal reflux using real-time MRI: a standardized approach to image acquisition and assessment

**DOI:** 10.1038/s41598-023-49776-w

**Published:** 2023-12-21

**Authors:** Lorenz Biggemann, Johannes Uhlig, Ulrike Streit, Omar Al-Bourini, Edris Wedi, Ahmad Amanzada, Volker Ellenrieder, Felix Rühlmann, Michael Ghadimi, Jens Frahm, Martin Uecker, Ali Seif Amir Hosseini

**Affiliations:** 1 Department of Diagnostic and Interventional Radiology, University Medical Center Göttingen, Göttingen, Germany; 2 Department of Radiology, Klinikum Rechts Der Isar, School of Medicine, Technical University of Munich, München, Germany; 3 Department of Gastroenterology and Gastrointestinal Oncology, University Medical Center Göttingen, Göttingen, Germany; 4 Department of Gastroenterology, Gastrointestinal Oncology and Interventional Endoscopy, Sana Klinikum, Offenbach, Germany; 5 Department of General, Visceral, and Paediatric Surgery, University Medical Center, Göttingen, Germany; 6 Max Planck Institute for Multidisciplinary Sciences, Göttingen, Germany; 7 Institute of Biomedical Imaging, Graz University of Technology, Graz, Austria; 8 Cluster of Excellence “Multiscale Bioimaging: From Molecular Machines to Networks of Excitable Cells” (MBExC), University of Göttingen, Göttingen, Germany

**Keywords:** Gastrointestinal diseases, Gastrointestinal system

## Abstract

This study aims to develop a standardized algorithm for gastroesophageal image acquisition and diagnostic assessment using real-time MRI. Patients with GERD symptoms undergoing real-time MRI of the esophagus and esophagogastric junction between 2015 and 2018 were included. A 10 ml bolus of pineapple juice served as an oral contrast agent. Patients performed Valsalva maneuver to provoke reflux and hiatal hernia. Systematic MRI assessment included visual presence of achalasia, fundoplication failure in patients with previous surgical fundoplication, gastroesophageal reflux, and hiatal hernia. A total of 184 patients (n = 92 female [50%], mean age 52.7 ± 15.8 years) completed MRI studies without adverse events at a mean examination time of 15 min. Gastroesophageal reflux was evident in n = 117 (63.6%), hiatal hernia in n = 95 (52.5%), and achalasia in 4 patients (2.2%). Hiatal hernia was observed more frequently in patients with reflux at rest (n = 67 vs. n = 6, *p* < 0.01) and during Valsalva maneuver (n = 87 vs. n = 8, *p* < 0.01). Real-time MRI visualized a morphologic correlate for recurring GERD symptoms in 20/22 patients (90%) after fundoplication procedure. In a large-scale single-center cohort of patients with GERD symptoms undergoing real-time MRI, visual correlates for clinical symptoms were evident in most cases. The proposed assessment algorithm could aid in wider-spread utilization of real-time MRI and provides a comprehensive approach to this novel imaging modality.

## Introduction

Real-time magnetic resonance imaging (MRI) is an imaging modality that combines high image quality with high temporal resolution of up to 20 ms per frame. The proposed technique relies on strongly undersampled data acquisition in combination with nonlinear inverse image reconstruction^1^. This novel imaging approach has shown promise in a wide spectrum of clinical applications, such as cardiovascular imaging, musculoskeletal imaging, as well as blood flow and cerebrospinal fluid dynamics^2^.

A specific clinical real-time MRI application is the visualization of deglutition and esophageal bolus transit for the evaluation of dysphagia and gastroesophageal reflux disease (GERD)^3–6^. In several studies, real-time MRI of the lower esophagus and esophagogastric junction (EGJ) has been evaluated for the detection of GERD, esophageal motility disorders, hiatal hernias, and the evaluation of the EGJ in patients after anti-reflux surgery using dynamic and anatomic imaging parameters^7–12^. So far, however, studies focused on single aspects of esophageal transit and pathologies and failed to provide a comprehensive and reproducible approach to real-time image acquisition and assessment.

This study aims to (1) develop a standardized real-time MRI acquisition protocol and (2) a pragmatic flow chart for image assessment that implements detection of gastroesophageal reflux, motility disorders and complications of fundoplication procedures.

## Materials and methods

### Study population

This single-center study was conducted between 2015 and 2018 in accordance with the Declaration of Helsinki and received prior approval by the ethics board of the University Medical Center Goettingen (Nr. 14/5/18). All patients gave written informed consent before MRI examinations. For inclusion in this study, patients were required to present with typical GERD symptoms for a minimum of 6 months at the outpatient clinic of either the Department of General, Visceral, and Pediatric Surgery or the Department of Gastroenterology and Gastrointestinal Oncology and Endocrinology, both at the University Medical Center Goettingen, Germany. General exclusion criteria were pacemakers/ICDs as well as MRI incompatible implants, such as surgically implanted anti reflux devices. Further exclusion criteria were allergy to pineapple and inability to swallow.

### Real-time MRI

Real-time MRI parameters with undersampled radial FLASH and a corresponding examination protocol for assessment of the esophagus and EGJ have been extensively described in previous studies^7,9,10,12^. Ultra-fast spin echo (HASTE) and balanced steady-state gradient echo (TRUFI) MRI sequences in axial and coronal planes served as planning sequences for sagittal real-time MRI acquisitions of the middle and lower esophagus and EGJ. Next followed a real-time acquisition of bolus transit through the lower esophagus and EGJ in coronal plane. These recordings were followed by real-time MRI acquisitions of the same planes during Valsalva maneuver. In case of insufficient visualization of the esophagus or EGJ, real-time MRI acquisitions were repeated with optimized plane orientation if necessary.

All real-time MRI films were acquired in a single plane using the subsequent parameters: TR = 2.12 ms, TE = 1.31 ms, flip angle 8°, FOV 256 × 256 mm^2^, in-plane resolution 1.5 × 1.5 mm^2^, slice thickness 8 mm. The temporal resolution was 40 ms per frame or 25 frames per second (fps). All real-time MRI acquisitions lasted for 700 frames or a total of 28 s.

A 10 mL bolus of pineapple juice served as an oral contrast agent due to its high manganese content. Therefore, no off-label use of gadolinium-based contrast agent was required.

**Figure 1:**
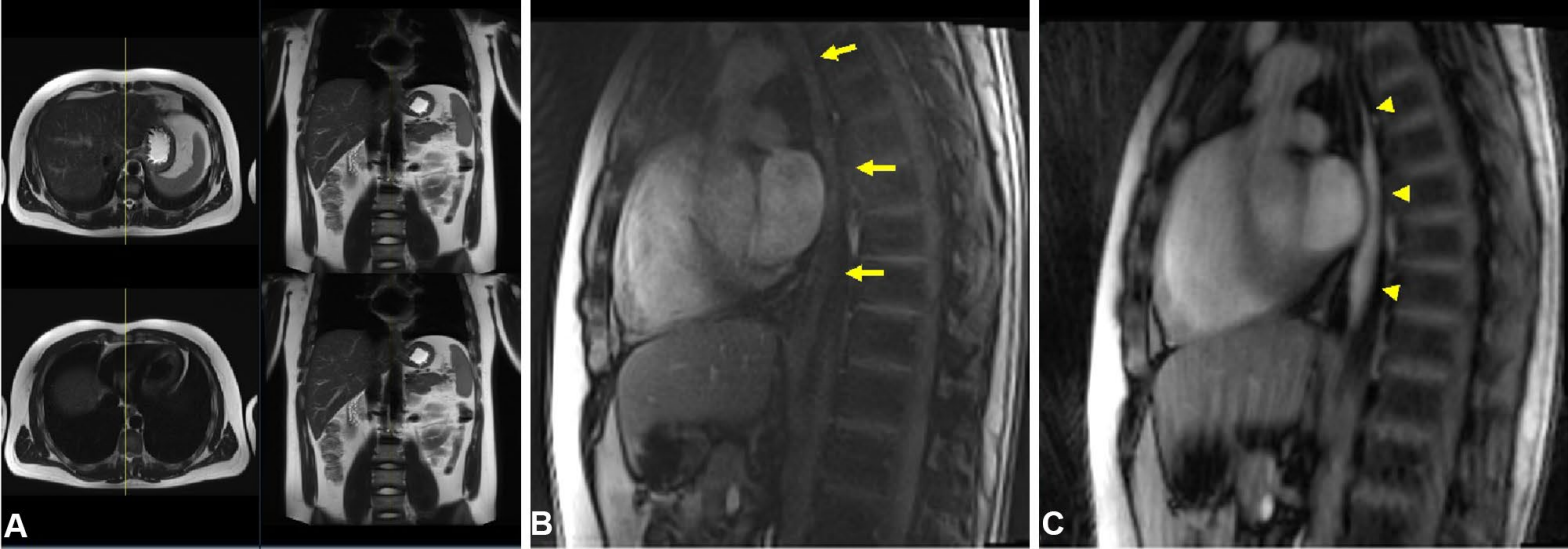
Definition of sagittal real-time MRI of the esophagus. (**A**) Axial and coronal HASTE planning images for sagittal real-time MRI. (**B**, **C**) Real-time MRI of the empty esophagus before bolus arrival (yellow arrows) and during bolus transit (yellow arrowheads).

**Figure 2:**
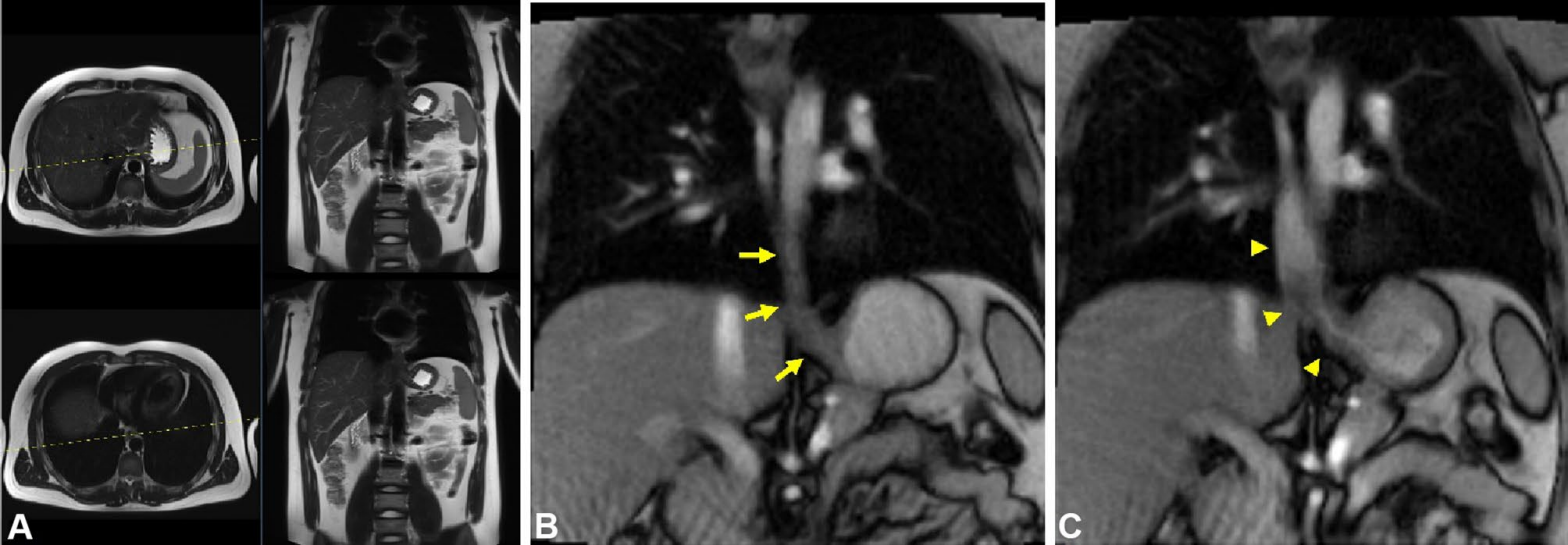
Definition of coronal real-time MRI of EGJ. (**A**) Axial and coronal HASTE planning images for coronal real-time MRI of EGJ. (**B**, **C**) Real-time MRI of the empty esophagus before bolus arrival (yellow arrows) and during bolus transit (yellow arrowheads).

Imaging examples highlighting the plane orientations are presented in Figs. 1 and 2. Further examples of plane orientation in patients with challenging EGJ anatomy are provided in Figures S1 and S2 of the supplemental material. The real-time MRI protocol of the esophagus and EGJ is summarized in Table 1.

**Table 1:** Real-time MRI protocol of the esophagus and EGJ.

Investigation	Sequence	Orientation	Slice thickness (mm)	Example
Planning	HASTE	axial	5	–
		coronal	5	–
	TRUFI	axial	5	–
		coronal	5	–
Bolus Transit	Real-time MRI	sagittal	8	Fig. 1
		coronal EGJ	8	Fig. 2
Valsalva maneuver	Real-time MRI	Sagittal	8	–
		coronal EJG	8	–
Optional Planes	Real-time MRI	axial EGJ	8	Figure S1

### MR image evaluation

Assessment of conventional MRI sequences and real-time MRI films were performed by two attending radiologists by consensus reading. Reader 1 (AS) was an attending radiologist with 11 years of experience in abdominal radiology and 4 years of experience in real-time MRI of the esophagus and EGJ. Reader 2 (LB) was an attending radiologist with 7 years of experience in abdominal radiology and 4 years of experience in real-time MRI of the esophagus and EGJ. Analyses were based on the manufacturer’s software (Syngo B17, Siemens Healthineers, Erlangen, Germany). In cases of disagreement, MRI films were reassessed by both readers by consensus reading.

The presence or absence of reflux under Valsalva maneuver and the presence of hiatal hernias were evaluated visually by both readers. Reflux was defined as the presence of visible fluid level or signal increase in the esophagus during Valsalva maneuver.

Based on the experience with real-time studies, both readers identified and defined the main steps of the image acquisition process and critical decision-making of real-time MRI film interpretation. Based on these main steps, a flow chart for an optimized, time-efficient real-time MRI protocol was developed by consensus by both readers (AS and LB).

### Statistical analysis

Continuous variables are given as mean ± standard deviation (SD), and categorical variables as absolute values and percentage. The chi-square and Wilcoxon rank-sum tests were used to evaluate distribution of categorical and continuous variables, respectively, across patient subgroups. All statistical analyses were performed using R version 4.2.1 and RStudio Version 2022.07.1. An alpha level of 0.05 was chosen to indicate statistical significance. All provided p-values are two-sided.

## Results

### Patient cohort

A total of n = 184 patients were included in this study. The mean patient age was 52.7 ± 15.8 years. Overall gender distribution of included patients was balanced with n = 92 female (50%) and n = 92 male (50%) patients (*p* = *0.36*). The median duration of symptoms was 30 months with an interquartile range of 48 months. A total of 30 patients did not provide a specific duration of GERD symptoms and estimated the symptoms being present “…for multiple years”. The mean was BMI of included patients was 26.8 ± 5.9 kg/m^2^. Patients with visible reflux had a higher mean BMI of 28.0 ± 6.1 kg/m^2^ compared to those without visible reflux (mean BMI 24.1 ± 3.5 kg/m^2^; *p* < *0.01*). Median scan time of real-time MRI was 15 min. The characteristics of the study population are summarized in Table 2.

**Table 2:** Patient cohort and real-time MRI parameters.

	Total	No reflux	Reflux	*p*-value
Patients	184	67	117	–
Age (year)	52.7 ± 15.8	44.6 ± 15.2	57.3 ± 14.3	< 0.01
Gender				
Female	92 (50.0%)	37 (55.2%)	55 (47.0%)	0.36
Male	92 (50.0%)	30 (44.8%)	62 (53.0%)	
BMI	26.8 ± 5.9	24.1 ± 3.5	28.0 ± 6.1	< 0.01
Spontaneous reflux	31 (16.8%)	–	31 (26.5%)	< *0.01*
Hiatal hernia				
Resting	73 (40.3%)	6 (9.1%)	67 (58.3%)	< *0.01*
Valsalva maneuver	95 (52.5%)	8 (12.3%)	87 (75.0%)	< *0.01*
Hernia size (cm)	3.4 ± 2.6	1.6 ± 1.6	3.6 ± 2.6	*0.01*
Sliding hernia	38 (21.0%)	6 (9.2%)	32 (27.6%)	*0.01*

All parameters are either presented as mean ± standard deviation or absolute numbers and percentages.

### Standardized real-time MRI assessment

**Figure 3:**
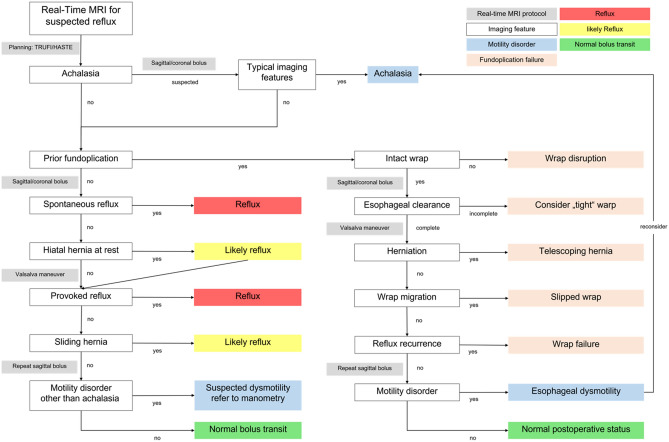
Diagnostic flow chart for real-time MRI of the esophagus and EGJ. In a first step, all real-time MRI films are evaluated for the presence of achalasia. If achalasia is confirmed, the study is finished, and no further real-time images should be acquired. Second, patients are evaluated for the presence or failure of prior fundoplication surgery. In case of prior anti-reflux surgery, real-time MRI films should be assessed for the presence of specific fundoplication failure patterns^10,17^. Otherwise, patients should be assessed for the presence of reflux and hiatal hernia confirming the presence of gastroesophageal reflux disease. In the absence of reflux provocation, the presence of sliding hiatal hernia during Valsalva maneuver should be considered suggestive of GERD. In cases lacking unequivocal reflux or signs of failure of anti-reflux surgery, esophageal motility disorders should be considered as a differential diagnosis especially in patients with incomplete or delayed esophageal clearance. Due to the lack of specific imaging parameters, these patients should be referred to additional high-resolution manometry.

Based on the expertise with real-time MRI of the esophagus and EGJ from earlier projects and the large-scale cohort available at University Medical Center Göttingen, a standardized real-time assessment was developed (see Fig. 3) that is applicable to both surgically naïve patients and those after surgical fundoplication.

Using this specific algorithm, the following pathologies were evident on real-time MRI:

Gastroesophageal reflux was present in 117/184 patients (63.6%). Spontaneous reflux after deglutition of pineapple juice without Valsalva maneuver was evident in n = 31/184 patients (26.5%). Patients without visible reflux on real-time MRI were significantly younger with a mean age of 44.6 ± 15.2 years compared to patients with visible reflux with a mean age of 57.3 ± 14.3 years (*p* < *0.01*). Hiatal hernia was evident on real-time MRI films in 95/184 patients (52.5%). Moreover, presence of hiatal hernia was significantly higher in patients with reflux at resting position (58.3% vs. 9.1%, *p* < *0.01*) and during Valsalva maneuver (12.3% vs. 75.0%, *p* < *0.01*) also resulting in a higher presence of sliding hernia (9.2% vs. 27.6%, *p* = *0.01*) in patients with visible reflux. Hiatal hernias were larger in patients with reflux on real-time MRI (3.6 ± 2.6 cm vs.1.6 ± 1.6 cm; *p* = *0.01*). A total 91 patients underwent combined 24-h pH-metry and impedance. Combined pH-metry and impedance was pathological in n = 67 patients (73.6%). Reflux was also detected by real-time MRI in 52/67 patients (77.6%). The majority of patients with normal 24-h pH-metry and impedance measurements also did not present reflux on real-time MRI (16/24; 67.7%). Four patients were diagnosed with achalasia (2.2%) on real-time MRI films, which were confirmed by manometry. Real-time MRI parameters are summarized in Table 2.

### Interpretability of anatomic real-time MRI parameters

The visual interpretability of MRI parameters was evaluated by both readers by consensus reading. Parameter interpretability was considered excellent with less than 5.0% non-interpretable cases for presence of hiatal hernia and sliding hernia (see Table 3).

**Table 3:** Assessability of anatomic and functional real-time MRI parameters.

	Total (n = 184)	No reflux (n = 67)	Reflux (n = 117)
Hiatal hernia			
Resting	3 (1.6%)	1 (1.5%)	2 (1.7%)
Valsalva maneuver	3 (1.6%)	2 (3.0%)	1 (0.9%)
Sliding hernia	3 (1.6%)	2 (3.0%)	1 (0.9%)

Presented are the number of cases which were ruled as not assessable by both readers for each parameter.

## Discussion

With technical advances and the advent of real-time MRI, physiological processes can be directly monitored at high temporal resolution. One application of real-time MRI is the visualization of deglutition and gastroesophageal reflux. In earlier studies, we have demonstrated the diagnostic capability of real-time MRI to assess hiatal hernias, gastroesophageal reflux, motility disorders, as well as complications after fundoplication^10–12^. However, we never provided a systematic approach to time-effective image acquisition and essential steps of image interpretation.

This study aims to provide a standardized algorithm for real-time MRI of the gastroesophageal junction and its diagnostic assessment. In total, a cohort of 184 patients with balanced gender distribution was included. Patients with visible reflux were older compared to those without.

Based on several years of expertise, we believe that the use of real-time MRI or another MRI technique with high spatiotemporal resolution should be considered especially in patients with suspected fundoplication failure. In the future, real-time MRI findings such as the presence of hiatal hernia or visible reflux may be auxiliary features that could aid establishing GERD diagnoses. However, further prospective studies are warranted to determine the diagnostic relevance of different MR imaging features. Furthermore, a standardized MRI protocol was developed and presented in this study, which allows for reproducible and consistent image acquisition. Following ultrafast HASTE and TRUFI planning sequences in axial and coronal planes, we propose real-time MRI sequences in sagittal orientation through the middle and lower esophagus and EGJ. Bolus transit should be evaluated in coronal orientation at rest and with Valsalva maneuver. Valsalva maneuver is an essential part of the real-time MRI protocol to optimize detection of sliding hiatal hernia. The here presented real-time MRI protocol was acquired at a median scan time of 15 min.

Because of the still limited availability of the applied real-time MRI technique, pertinent sequences might be substituted for other high-temporal resolution steady-state-free-precession (SSFP) MRI sequences such as accelerated versions of FLASH and TRUFI in other institutions. Based on our expertise, we believe that a temporal resolution of 4–6 frames per second should be sufficient to detect relevant EGJ pathologies.

In our study, pineapple juice served as oral contrast agent and demonstrated excellent visualization of the oral bolus. Utilization of pineapple juice yields several benefits, such as no off-label use of GBCA, high patient acceptance and a low risk of allergic reactions. Alternatively, agave syrup or a commercially produced mix of different fruit juices could be used as an oral contrast agent^13,14^.

In our institution, the proposed protocol was implemented both in treatment-naïve patients and those with prior anti-reflux surgery with robust results, allowing for visualization of the fundoplication wrap. While barium esophagram and upper gastrointestinal series are still routinely performed in clinical practice, they are no longer recommended for the diagnosis of GERD in current consensus guidelines^15,16^. Real-time MRI offers improved soft tissue resolution not only of the EGJ, but also of the surrounding structures without radiation exposure or invasive probe placement, which is particularly relevant in younger patients. Moreover, visualization of hiatal hernia or fundoplication failure by real-time MRI may assist in the planning and preparation of endoscopic and surgical procedures.

Further, this study developed a standardized clinical flow chart for real-time MR image assessment based on the experiences published in earlier studies^7–12^. This assessment flow chart solely relies on visual radiological assessment of esophageal and EGJ pathologies. One critical branching point is the presence of achalasia. If a patient with achalasia is diagnosed, no further diagnostic is necessary from our perspective. Next downstream, the clinical context of prior fundoplication surgery is of importance for further branching into fundoplication-associated pathologies and those in treatment-naïve patients. In our patient cohort, pathologies that could explain GERD symptoms were detected in the majority of patients, thus underlining the clinical applicability of the presented diagnostic flow chart. In general, most real-time MRI parameters had good overall radiological accessibility, thus corroborating the robustness of real-time MRI for evaluation of GERD.

The here presented diagnostic flow chart also incorporates relevant diagnoses as outlined by the most recent Chicago and Lyon classifications currently used for diagnosis of GERD patients in gastroenterological clinical practice. With further updates of these classifications, real-time MRI protocols might be adapted accordingly.

Of note, additional real-time MRI parameters were quantified in this cohort, such as the sphincter-transit-time or the esophagus-fundus-angle. However, most of these parameters did not correlate with visually detected pathologies. Thus, the clinical relevance of time-consuming real-time MRI image quantification remains questionable, particularly given the diagnostic flow chart presented in our study that relies on visual radiological assessment.

This study is not devoid of limitations. First, no cohort of participants without GERD symptoms was available, and thus no reference real-time MRI standard could be established. Moreover, this study did not employ a standardized GERD questionnaire to measure symptom intensity. This might limit the performance of our diagnostic flow chart, for example, if low-grade gastroesophageal reflux is detectable in participants without GERD symptoms. Further, the evaluation of other esophageal motility disorders than achalasia is still limited due to the lack of specific imaging parameters^11^. In addition, the accuracy of this diagnostic flow chart still has to be confirmed in upcoming studies. These studies should employ standardized questionnaires such as GERD-HRQL to quantify symptom intensity. Finally, this study evaluates a heterogeneous GERD patient cohort, i.e. those with and without surgical fundoplication. While this may aid in designing a more generalizable and clinically robust diagnostic MRI flow chart, underlying GERD symptoms will have different determinants, with potential limitation for the interpretability of individual MR studies. Additional information on specific GERD patient cohort are provided in earlier publications by this research group^7,9–12^.

For future directions, we aim to provide a standardized DICOM dataset of patients undergoing real-time MRI with annotated pathologies and the respective diagnostic flow chart pathway. This could further enable other institutions to train their personnel and to establish EGJ MR imaging in clinical practice.

## Conclusion

In conclusion, real-time MRI of the esophagus and esophagogastric junction holds diagnostic promise for a broad spectrum of patients presenting with GERD symptoms. An optimized real-time MRI acquisition protocol yields robust images within a short examination time and without the need for off-label GBCA administration. Utilizing a diagnostic flow chart for visual real-time MRI assessment, relevant pathologies can be detected in the majority of examined patients.

The here presented standardized methodology could aid in further clinical implementation of MRI with high temporal resolution in GERD patients in other institutions.

### Supplementary Information

Supplementary Information 1.

Supplementary Information 2.

Supplementary Video 1.

Supplementary Video 2.

## Data Availability

Datasets used/and or analyzed in the current study available from the corresponding author upon reasonable request.

## Abbreviations

BMI: Body mass index
EGJ: Esophagogastric junction
FOV: Field of view
GBCA: Gadolinium based contrast agents
GERD: Gastroesophageal reflux disease
HRQL: Health-related quality of life
SSFP: Steady-state free precession
