# Supplementary material for: Visualization of deglutition and gastroesophageal reflux using real-time MRI: a standardized approach to image acquisition and assessment
Source: Sci Rep. 2023 Dec 21;13:22854. doi: 10.1038/s41598-023-49776-w (PMC10739804; doi:10.1038/s41598-023-49776-w)
Supplement: Supplementary file 1 — Supplementary Information 1. [file 41598_2023_49776_MOESM1_ESM.docx]

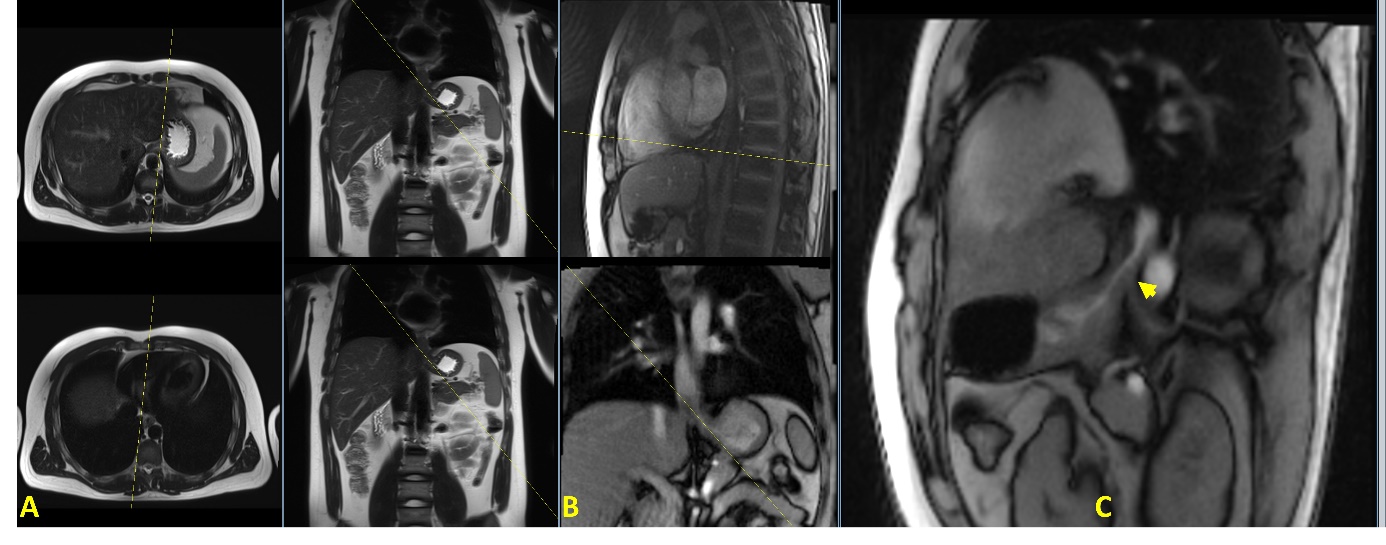


**Figure S1: Definition of para-axial real-time MRI of LES.** In case of insufficient visualization of the LES in sagittal and coronal real-time MRI planes, a para-axial LES plane was established. (A) Axial and coronal HASTE planning images for the para-axial LES plane. (B) Previously acquired real-time MRI planes for optimization of the (C) para-axial plane orientation.
