# Supplementary material for: Visualization of deglutition and gastroesophageal reflux using real-time MRI: a standardized approach to image acquisition and assessment
Source: Sci Rep. 2023 Dec 21;13:22854. doi: 10.1038/s41598-023-49776-w (PMC10739804; doi:10.1038/s41598-023-49776-w)
Supplement: Supplementary file 2 — Supplementary Information 2. [file 41598_2023_49776_MOESM2_ESM.docx]

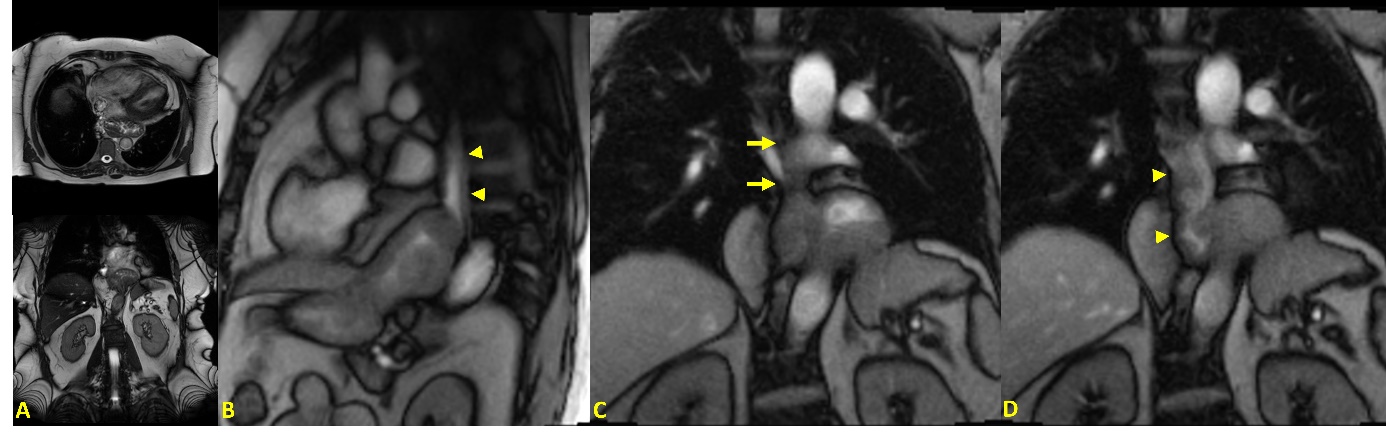


**Figure S2: Visualization of the LES in a patient with challenging anatomy.** (A) Axial and coronal HASTE images reveal a large hiatal hernia. (B) Due to the alterned anatomy, real-time MRI depicts only a short segment of the distal esophagus during bolus transit (arrow heads). (C) In coronal planes the empty esophagus can only vaguely be recognised at rest due to partial volume effects (arrows). (D) After plane optimization employing previous real-time aquisitions the lowest segment of the esophagus and the LES could successfully be visualized during bolus transit (arrowheads).
